# Crystal structure and Hirshfeld surface analysis of (3a*SR*,6*RS*,6a*SR*,7*RS*,11b*SR*,11c*RS*)-2,2-dibenzyl-2,3,6a,11c-tetra­hydro-1*H*,6*H*,7*H*-3a,6:7,11b-di­epoxy­dibenzo[*de*,*h*]isoquinolin-2-ium tri­fluoro­methane­sulfonate

**DOI:** 10.1107/S2056989021010173

**Published:** 2021-10-08

**Authors:** Zeliha Atioğlu, Mehmet Akkurt, Gunay Z. Mammadova, Sixberth Mlowe

**Affiliations:** a Department of Aircraft Electrics and Electronics, School of Applied Sciences, Cappadocia University, Mustafapaşa, 50420 Ürgüp, Nevşehir, Turkey; b Department of Physics, Faculty of Sciences, Erciyes University, 38039 Kayseri, Turkey; c Department of Chemistry, Baku State University, Z. Khalilov str. 23, AZ, 1148 Baku, Azerbaijan; d University of Dar es Salaam, Dar es Salaam University College of Education, Department of Chemistry, PO Box 2329, Dar es Salaam, Tanzania

**Keywords:** crystal structure, tetra­hydro­furan ring, piperidine ring, C—H⋯O hydrogen bonds, Hirshfeld surface analysis, IMDAF reaction, Diels–Alder reaction

## Abstract

In the cation of the title salt, C_30_H_28_NO_2_^+^·CF_3_O_3_S^−^, the four tetra­hydro­furan rings adopt envelope conformations. In the crystal, pairs of cations are linked by dimeric C—H⋯O hydrogen bonds, forming two *R* ^2^_2_(6) ring motifs parallel to the (001) plane. The cations and anions are connected by further C—H⋯O hydrogen bonds, forming a three-dimensional network structure. Hirshfeld surface analysis indicates that the most important contributions to the crystal packing are from H⋯H (47.6%), C⋯H/H⋯C (20.6%), O⋯H/H⋯O (18.0%) and F⋯H/H⋯F (9.9%) inter­actions.

## Abstract 2

In the crystal, dimeric C—H⋯O hydrogen bonds connect pairs of cations, producing two 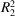 (6) ring motifs parallel to the (001) plane. Inter­molecular C—H⋯O hydrogen connections connect the cations and anions, producing a three-dimensional network.

## Chemical context

Intra­molecular Diels–Alder reactions (Krishna *et al.*, 2021 ▸) are powerful tools in the arsenal of modern organic chemistry. In particular, the IMDAF cyclo­addition (the intra­molecular furan Diels–Alder reaction) based on renewable starting materials (*e.g*. furfural, furfuryl alcohol, *etc*.), is frequently used in natural product synthesis and in many other practically useful applications (for reviews on the topic, see: Zubkov *et al.*, 2005 ▸; Takao *et al.*, 2005 ▸; Juhl *et al.*, 2009 ▸; Padwa *et al.*, 2013 ▸; Parvatkar *et al.*, 2014 ▸). Cascade sequences including two or more successive [4 + 2] cyclo­addition steps to furan moieties are less known because of difficulties in accessing the starting materials. However, these tandem strategies open up an easy way for the construction of polyfunctional naphthalene derivatives, which can be obtained in one synthetic step. At the same time, it becomes possible to create four or more chiral centres in one synthetic stage with exceptional chemo-, regio- and diastereoselectivity (Criado *et al.*, 2010 ▸, 2013 ▸; Zubkov *et al.*, 2012 ▸, 2014 ▸). Previously, it was shown that the [4 + 2] cyclo­addition of *bis*-furyldienes with derivatives of maleic acid, esters of acetyl­ene di­carb­oxy­lic acid or hexa­fluoro-2-butyne proceeds in all cases with excellent diastereo- and chemoselectivity, and leads, depending on the temperature, to annelated di­epoxy­naphthalenes of the ‘domino’ or ‘pincer’ type (Borisova *et al.*, 2018*a* ▸,*b* ▸).

**Figure 1:**
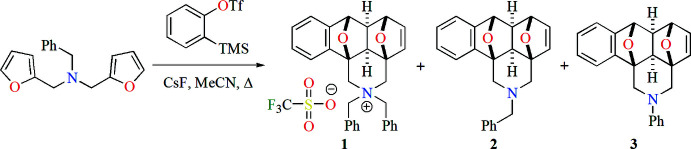
Synthesis scheme of the title compound **1** and its by-products.

In order to expand the limits of the applicability of the IMDAF strategy, during the current study we tested de­hydro­benzene generated *in situ* in the role of a dienophile. It was found that *N*-benzyl­difurfuryl­amine under the action of de­hydro­benzene forms a multicomponent mixture, from which three major components (**1**–**3**) were isolated using column chromatography (Fig. 1 ▸). Compound **1**, the most inter­esting from a chemical point of view, was chosen for structural analysis using diffraction data. 
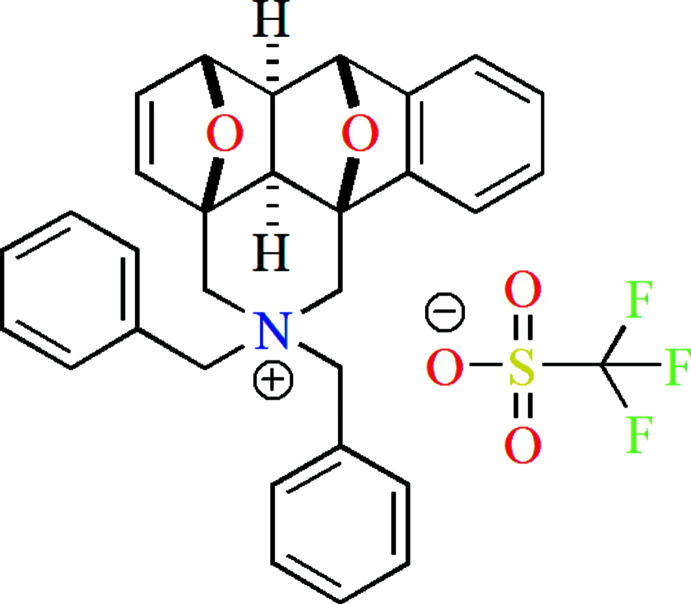


In general, non-covalent inter­actions such as hydrogen bonding, ionic and π-inter­actions play critical roles in synthesis and catalysis, as well as in the organization of the supra­molecular structures as a result of their significant contribution to the self-assembly process (Gurbanov *et al.*, 2020*a* ▸,*b* ▸; Khalilov *et al.*, 2018*a* ▸,*b* ▸; Ma *et al.*, 2017*a* ▸,*b* ▸, 2020 ▸, 2021 ▸; Mahmudov *et al.*, 2012 ▸, 2020 ▸; Mizar *et al.*, 2012 ▸). Thus, the inter­play of non-covalent inter­actions has an impact on solubility (Shixaliyev *et al.*, 2019 ▸) and other functional properties of **1**.

## Structural commentary

**Figure 2:**
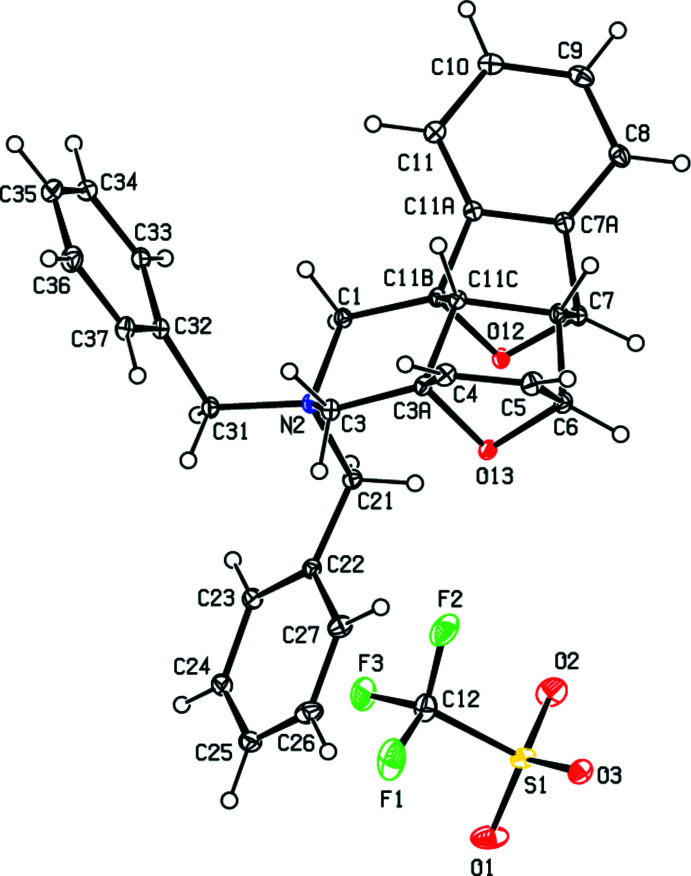
The asymmetric unit of the title salt **1** with displacement ellipsoids for the non-hydrogen atoms drawn at the 30% probability level.

In the cation (C_30_H_28_NO_2_^+^) of the title salt **1** (Fig. 2 ▸), the tetra­hydro­furan rings (O12/C7/C6*A*/C11*C*/C11*B*, O12/C7/C7*A*/C11*A*/C11*B*, O13/C3*A*/C4/C5/C6 and O13/C3*A*/C11*C*/C6*A*/C6) adopt envelope conformations with the following puckering parameters (Cremer & Pople, 1975 ▸): *Q*(2) = 0.5504 (8) Å, φ(2) = 181.08 (9)°, *Q*(2) = 0.5474 (9) Å, φ(2) = 0.24 (10)°, *Q*(2) = 0.5260 (9) Å, φ(2) = 1.91 (11)° and *Q*(2) = 0.5610 (9) Å, φ(2) = 175.78 (9)°, respectively. The mol­ecular conformation of the cation is stabilized by weak intra­molecular C21—H21*B*⋯O12 and C21—H21*B*⋯O13 contacts (Table 1 ▸). The piperidine ring (N2/C1/C11*B*/C11*C*/C3*A*/C3) in the cation exhibits a chair conformation [puckering parameters are Q_T_ = 0.4871 (9) Å, θ = 175.22 (11)° and φ = 281.2 (12)°]. The benzene ring (C7*A*/C8–C11/C11*A*) fused with the central tetra­hydro­furan ring makes dihedral angles of 53.43 (5) and 58.64 (5)°, respectively, with the C22–C27 and C32–C37 phenyl rings of the benzyl groups attached to the N atom. These phenyl rings make a dihedral angle of 73.81 (5)° with each other.

## Supra­molecular features and Hirshfeld surface analysis

**Figure 3:**
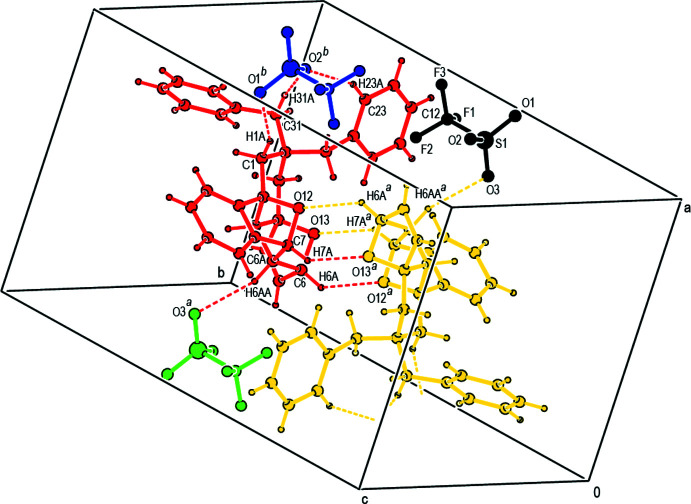
A general view of the inter­molecular C—H⋯O hydrogen bonds (depicted by dashed lines) in the unit cell of the title salt **1**. [Symmetry codes: (*a*) 1 − *x*, 1 − *y*, 1 − *z*; (*b*) 2 − *x*, 1 − *y*, 1 − *z*].

**Figure 4:**
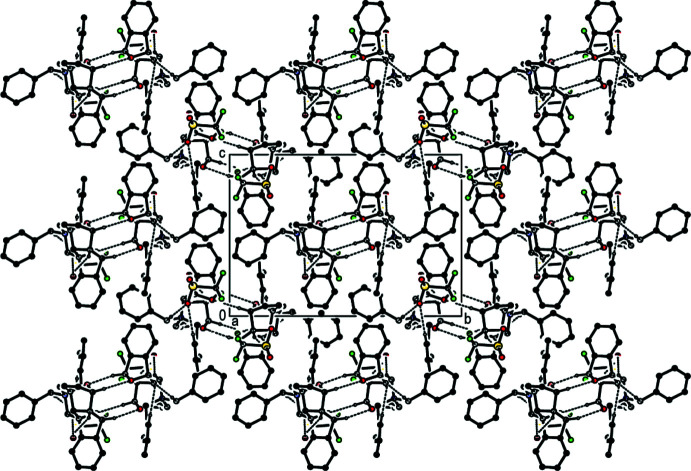
Packing of the title salt **1** viewed along the *a* axis direction with C—H⋯O hydrogen bonds shown as dashed lines.

In the crystal, pairs of cations are linked by dimeric C6—H6*A*⋯O12^*ii*^ and C7—H7*A*⋯O13^ii^ hydrogen bonds [symmetry code: (ii) −*x* + 1, −*y* + 1, −*z* + 1], forming two 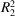 (6) ring motifs (Bernstein *et al.*, 1995 ▸) parallel to the (001) plane (Table 1 ▸; Fig. 3 ▸). Furthermore, the cations and anions are connected by inter­molecular C1—H1*A*⋯O1^i^, C6—H6*A*⋯O12^ii^, C6*A*—H6*AA*⋯O3^ii^, C7—H7*A*⋯O13^ii^, C31—H31*A*⋯O2^i^ and C9—H9*A*⋯*Cg*10^iii^ hydrogen bonds, forming a three-dimensional network (Table 1 ▸; Figs. 4 ▸, 5 ▸ and 6 ▸).

**Figure 7:**
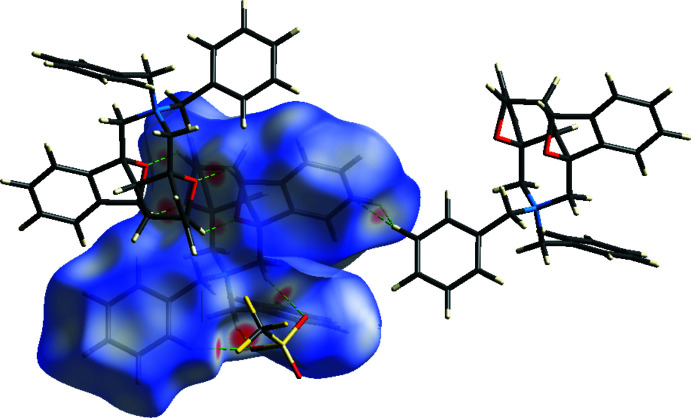
Hirshfeld surface of the title mol­ecule **1** mapped over *d*_norm_.

**Figure 8:**
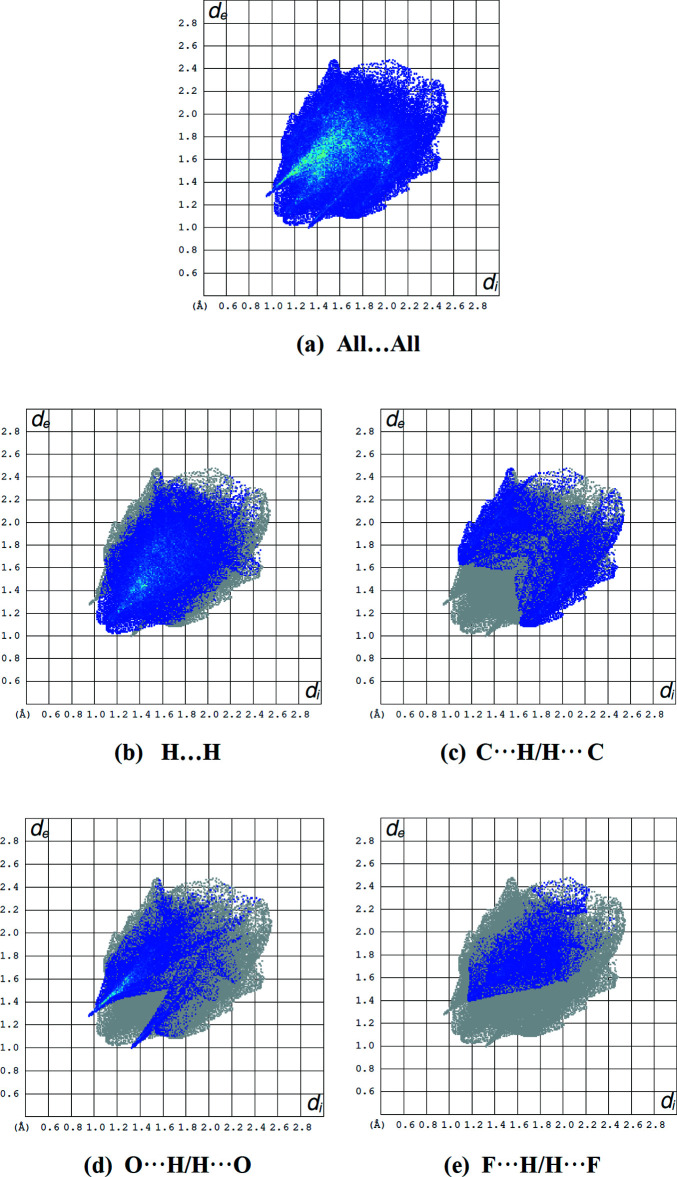
Fingerprint plots showing (*a*) all inter­molecular inter­actions and delineated into (*b*) H⋯H, (*c*) C⋯H/H⋯C, (*d*) O⋯H/H⋯O and (*e*) F⋯H/H⋯F contacts.

The inter­molecular inter­actions (Table 2 ▸) were qu­anti­fied and displayed using *CrystalExplorer17.5* (Turner *et al.*, 2017 ▸). Fig. 7 ▸ shows the Hirshfeld surface plotted over *d*_norm_ in the range −0.2715 to 1.3713 a.u. where C—H⋯O inter­actions are shown as red dots. The overall two-dimensional fingerprint plot, as well as those delineated into the main contacts, are shown in Fig. 8 ▸. The H⋯H (Fig. 8 ▸ *b*) inter­actions constitute the primary factor in the crystal packing, with C⋯H/H⋯C (Fig. 8 ▸ *c*), O⋯H/H⋯O (Fig. 8 ▸ *d*) and F⋯H/H⋯F (Fig. 8 ▸ *e*) inter­actions constituting the next stronger contributions. Numerical values of these inter­actions together with other percentage contributions of weaker inter­actions are compiled in Table 3 ▸.

## Database survey

A search of the Cambridge Structural Database (CSD version 5.40, update of September 2019; Groom *et al.*, 2016 ▸) for structures having an ep­oxy­iso­indole moiety gave ten hits that closely resemble the title salt, *viz.* IQOTOA (Mertsalov *et al.*, 2021*a* ▸), OMUTAU (Mertsalov *et al.*, 2021*b* ▸), OMEMAX (Mertsalov *et al.*, 2021*c* ▸), IMUBIE (Mertsalov *et al.*, 2021*a* ▸), AGONUH (Temel *et al.*, 2013 ▸), TIJMIK (Demircan *et al.*, 2013 ▸), YAXCIL (Temel *et al.*, 2012 ▸), UPAQEI (Koşar *et al.*, 2011 ▸), ERIVIL (Temel *et al.*, 2011 ▸) and MIGTIG (Koşar *et al.*, 2007 ▸).

IQOTOA, OMUTAU and OMEMAX each crystallize with two mol­ecules in the asymmetric unit. In the crystal, mol­ecule pairs generate centrosymmetric rings with 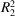 (8) motifs linked by C—H⋯O hydrogen bonds. These pairs of mol­ecules form a tetra­meric supra­molecular motif, leading to mol­ecular layers parallel to the (100) plane by C—H⋯π and C—Br⋯π inter­actions. Inter­layer van der Waals and inter­halogen inter­actions stabilize the mol­ecular packing. In the crystal of OMUTAU, strong inter­molecular O—H⋯O hydrogen bonds and weak inter­molecular C—H⋯O contacts link the mol­ecules, forming a three-dimensional network. In addition, weak π–π stacking inter­actions between the pyrrolidine rings of the nine-membered groups of mol­ecules are observed. In the crystal of OMEMAX, mol­ecules are linked by weak C—H⋯O hydrogen bonds, forming sheets lying parallel to the (002) plane. These sheets are connected only by weak van der Waals inter­actions. In the crystal of IMUBIE, the mol­ecules are linked into dimers by pairs of C—H⋯O hydrogen bonds, thus generating 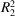 (18) rings. The crystal packing is dominated by H⋯H, Br⋯H, H⋯π and Br⋯π inter­actions. In the crystal structures of IQOTOA, OMUTAU, OMEMAX, AGONUH, TIJMIK, YAXCIL, UPAQEI and ERIVIL, the mol­ecules are predominantly linked by C—H⋯O hydrogen bonds describing different hydrogen-bonding pattern connectivities. In the crystal of AGONUH, the mol­ecules are connected into zigzag chains running along the *b-*axis direction. In TIJMIK, two types of C—H⋯O hydrogen bond motifs are found, *viz. R* ^2^_2_(20) and 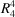 (26) rings, with adjacent rings running parallel to the *ac* plane. Additionally, C—H⋯O hydrogen bonds form a *C*(6) chain, linking the mol­ecules along the *b*-axis direction. In the crystal of ERIVIL, mol­ecules are connected into 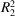 (8) and 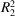 (14) rings along the *b* axis. In MIGTIG, the mol­ecules are linked only by weak van der Waals inter­actions.

## Synthesis and crystallization

**(3a** ***SR*** **,6** ***RS*** **,6a** ***SR***,**7** ***RS***,**11b** ***SR***,**11c** ***RS)*** **-2,2-Dibenzyl-2,3,6a,11c-tetra­hydro-1** ***H*** **,6** ***H*** **,7** ***H*** **-3a,6:7,11b-di­epoxy­dibenzo[** ***de***, ***h*** **]isoquinolin-2-ium tri­fluoro­methane­sulfonate (1)**

Cesium fluoride (CsF) (1.7 g, 0.011 mol) was added to benzyl­bis­(furan-2-ylmeth­yl)amine (0.0022 mol) dissolved in dry CH_3_CN (20 ml). Then an equivalent of 2-(tri­methyl­sil­yl)phenyl tri­fluoro­methane­sulfonate (0.54 ml, 0.022 mol) was added to the solution under an argon atmosphere. The mixture was refluxed for 4 h (TLC control, Sorbfil plates for thin-layer chromatography, EtOAc:hexane, 1:3). After one more portion of 2-(tri­methyl­sil­yl)phenyl tri­fluoro­methane­sulfonate (0.27 mL, 0.011 mol) and CsF (1.7 g, 0.011 mol) had been added to the mixture, all procedures were repeated. After the mixture was cooled to room temperature, the CsF was filtered off through a thin layer of SiO_2_, and the resulting solution was concentrated under reduced pressure. The residue (yellow oil) turned out to be a multicomponent mixture. It was separated using column chromatography on silica gel. The least mobile fraction represented the target product, **1**. In addition, two by-products **2** (12%) and **3** (17%) were isolated. Single crystals of **1** were obtained by slow crystallization from ethyl acetate.

## Refinement details

Crystal data, data collection and structure refinement details are summarized in Table 4 ▸. All C-bound H atoms were placed at calculated positions using a riding model, with C—H = 0.95–1.00 Å, and with *U*_iso_(H) = 1.2*U*_eq_(C). Five reflections (011, 101, 020, 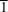 01 and 110), which were obscured by the beam stop as well as eight outliers (021, 111, 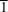 1 12, 218, 610, 143, 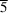 72 and 581) were omitted during the final cycle of refinement.

## Supplementary Material

Crystal structure: contains datablock(s) I. DOI: 10.1107/S2056989021010173/wm5617sup1.cif

Structure factors: contains datablock(s) I. DOI: 10.1107/S2056989021010173/wm5617Isup2.hkl

Additional supporting information:  crystallographic information; 3D view; checkCIF report

## Figures and Tables

**Figure 5:**
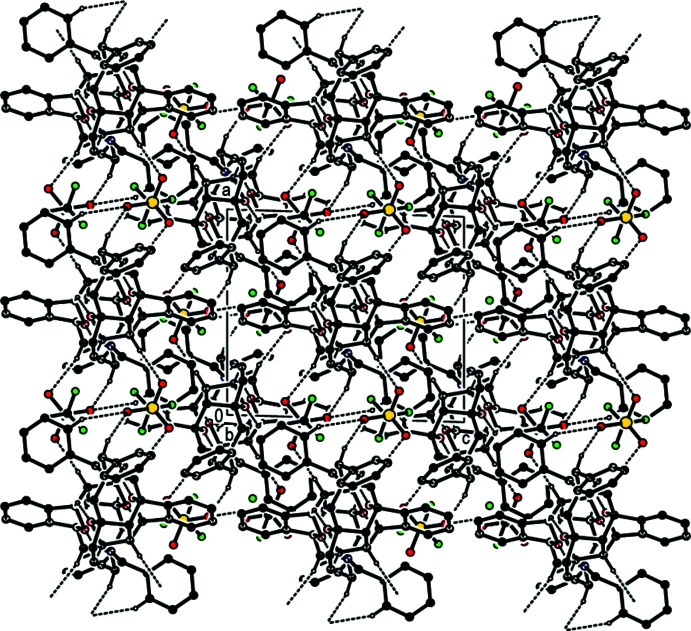
Packing of the title salt **1** viewed along the *b-*axis direction with C—H⋯O hydrogen bonds shown as dashed lines.

**Figure 6:**
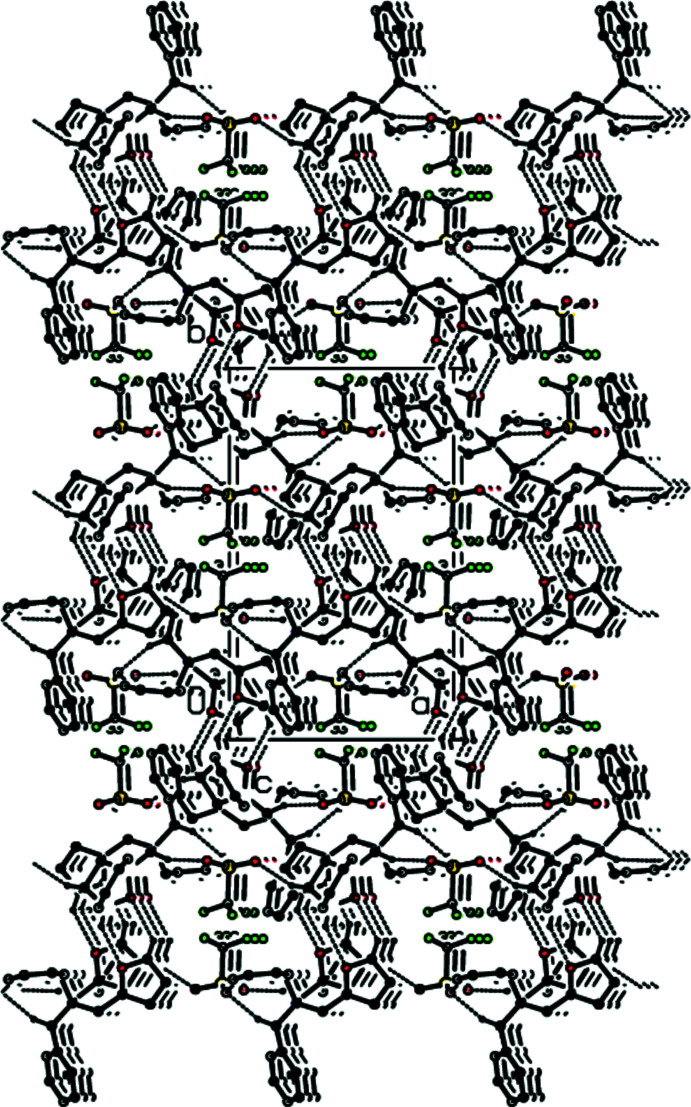
Packing of the title salt **1** viewed along the *c-*axis direction with C—H⋯O hydrogen bonds shown as dashed lines.

**Table 1:** Hydrogen-bond geometry (Å, °) *Cg*10 is the centroid of the C32–C37 ring.

*D*—H⋯*A*	*D*—H	H⋯*A*	*D*⋯*A*	*D*—H⋯*A*
C1—H1*A*⋯O1^i^	0.99	2.49	3.4043 (13)	154
C6—H6*A*⋯O12^ii^	1.00	2.52	3.3040 (11)	135
C6*A*—H6*AA*⋯O3^ii^	1.00	2.50	3.4407 (12)	157
C7—H7*A*⋯O13^ii^	1.00	2.41	3.2563 (11)	142
C21—H21*B*⋯O12	0.99	2.33	2.9151 (11)	117
C21—H21*B*⋯O13	0.99	2.35	3.0974 (11)	132
C31—H31*A*⋯O2^i^	0.99	2.33	3.2751 (13)	159
C9—H9*A*⋯*Cg*10^iii^	0.95	2.71	3.2958 (11)	121

Symmetry codes: (i) -x+2, -y+1, -z+1; (ii) -x+1, -y+1, -z+1; (iii) x-{\script{1\over 2}}, -y+{\script{3\over 2}}, z+{\script{1\over 2}}.

**Table 2:** Summary of short inter­atomic contacts (Å) in the title salt **1**

Contact	Distance	Symmetry operation
H7*A*⋯O13	2.41	1 − *x*, 1 − *y*, 1 − *z*
H4*A*⋯H11*A*	2.34	−{1\over 2} + *x*, {3\over 2} − *y*, −{1\over 2} + *z*
H31*A*⋯O2	2.33	2 − *x*, 1 − *y*, 1 − *z*
H37*A*⋯O3	2.65	{3\over 2} − *x*, {1\over 2} + *y*, {1\over 2} − *z*
H6*AA*⋯O3	2.50	1 − *x*, 1 − *y*, 1 − *z*
H9*A*⋯C8	3.01	1 − *x*, 1 − *y*, 2 − *z*
H1*B*⋯H24*A*	2.60	−{1\over 2} + *x*, {3\over 2} − *y*, {1\over 2} + *z*
H10*A*⋯H26*A*	2.31	*x*, *y*, 1 + *z*
C24⋯F1	3.202	*x*, *y*, *z*
C25⋯H36*A*	3.50	{3\over 2} − *x*, −{1\over 2} + *y*, {1\over 2} − *z*
H5*A*⋯H23*A*	2.53	−1 + *x*, *y*, *z*
H10*A*⋯O2	2.91	{3\over 2} − *x*, {1\over 2} + *y*, {1\over 2} − *z*

**Table 3:** Percentage contributions of inter­atomic contacts to the Hirshfeld surface of the title salt **1**

Contact	Percentage contribution
H⋯H	47.6
C⋯H/H⋯C	20.6
O⋯H/H⋯O	18.0
F⋯H/H⋯F	9.9
F⋯C/C⋯F	2.2
C⋯C	1.0
O⋯C/C⋯O	0.4
F⋯O/O⋯F	0.1

**Table 4:** Experimental details

Crystal data	
Chemical formula	C_30_H_28_NO_2_^+^·CF_3_O_3_S^−^
*M*_r_	583.60
Crystal system, space group	Monoclinic, *P*2_1_/*n*
Temperature (K)	100
*a*, *b*, *c* (Å)	11.1507 (9), 18.4653 (15), 12.8519 (10)
β (°)	91.786 (4)
*V* (Å^3^)	2644.9 (4)
*Z*	4
Radiation type	Mo *K*α
μ (mm^−1^)	0.19
Crystal size (mm)	0.40 × 0.32 × 0.16

Data collection	
Diffractometer	Bruker *KAPPA* APEXII area-detector diffractometer
Absorption correction	Multi-scan (*SADABS*; (Bruker, 2013 ▸)
*T*_min_, *T*_max_	0.924, 0.971
No. of measured, independent and observed [*I* > 2σ(*I*)] reflections	101039, 11684, 9164
*R*_int_	0.038
(sin θ/λ)_max_ (Å^−1^)	0.809

Refinement	
*R*[*F* ^2^ > 2σ(*F* ^2^)], *wR*(*F* ^2^), *S*	0.039, 0.109, 1.04
No. of reflections	11684
No. of parameters	370
H-atom treatment	H-atom parameters constrained
Δρ_max_, Δρ_min_ (e Å^−3^)	0.56, −0.32

Computer programs: *APEX2* and *SAINT* (Bruker, 2013 ▸), *SHELXT* (Sheldrick, 2015*a* ▸), *SHELXL* (Sheldrick, 2015*b* ▸), *ORTEP-3 for Windows* (Farrugia, 2012 ▸) and *PLATON* (Spek, 2020 ▸).

## supplementary crystallographic information

### Crystal data

**Table:**

C_30_H_28_NO_2_^+^·CF_3_O_3_S^−^	*F*(000) = 1216
*M**_r_* = 583.60	*D*_x_ = 1.466 Mg m^−^^3^
Monoclinic, *P*2_1_/*n*	Mo *K*α radiation, λ = 0.71073 Å
*a* = 11.1507 (9) Å	Cell parameters from 9807 reflections
*b* = 18.4653 (15) Å	θ = 2.7–34.6°
*c* = 12.8519 (10) Å	µ = 0.19 mm^−^^1^
β = 91.786 (4)°	*T* = 100 K
*V* = 2644.9 (4) Å^3^	Fragment, colourless
*Z* = 4	0.40 × 0.32 × 0.16 mm

### Data collection

**Table:**

Bruker KAPPA APEXII area-detector diffractometer	9164 reflections with *I* > 2σ(*I*)
φ and ω scans	*R*_int_ = 0.038
Absorption correction: multi-scan (*SADABS*; (Bruker, 2013)	θ_max_ = 35.1°, θ_min_ = 3.3°
*T*_min_ = 0.924, *T*_max_ = 0.971	*h* = −18→18
101039 measured reflections	*k* = −29→29
11684 independent reflections	*l* = −20→20

### Refinement

**Table:**

Refinement on *F*^2^	0 restraints
Least-squares matrix: full	Hydrogen site location: inferred from neighbouring sites
*R*[*F*^2^ > 2σ(*F*^2^)] = 0.039	H-atom parameters constrained
*wR*(*F*^2^) = 0.109	*w* = 1/[σ^2^(*F*_o_^2^) + (0.0527*P*)^2^ + 0.8665*P*] where *P* = (*F*_o_^2^ + 2*F*_c_^2^)/3
*S* = 1.04	(Δ/σ)_max_ = 0.001
11684 reflections	Δρ_max_ = 0.56 e Å^−^^3^
370 parameters	Δρ_min_ = −0.32 e Å^−^^3^

### Special details

**Table:**

Geometry. All esds (except the esd in the dihedral angle between two l.s. planes) are estimated using the full covariance matrix. The cell esds are taken into account individually in the estimation of esds in distances, angles and torsion angles; correlations between esds in cell parameters are only used when they are defined by crystal symmetry. An approximate (isotropic) treatment of cell esds is used for estimating esds involving l.s. planes.

### Fractional atomic coordinates and isotropic or equivalent isotropic displacement parameters (Å^2^)

**Table:**

	*x*	*y*	*z*	*U*_iso_*/*U*_eq_	
C1	0.67175 (8)	0.69811 (5)	0.60647 (6)	0.01270 (14)	
H1A	0.747874	0.677220	0.634517	0.015*	
H1B	0.660815	0.745773	0.640139	0.015*	
C3	0.56390 (8)	0.73115 (5)	0.43738 (7)	0.01317 (14)	
H3A	0.547400	0.782451	0.454167	0.016*	
H3B	0.572480	0.727711	0.361102	0.016*	
C3A	0.45754 (7)	0.68617 (5)	0.46733 (7)	0.01244 (13)	
C4	0.33252 (8)	0.71172 (5)	0.43326 (7)	0.01597 (15)	
H4A	0.308749	0.759245	0.413203	0.019*	
C5	0.26370 (8)	0.65278 (5)	0.43772 (8)	0.01802 (16)	
H5A	0.179772	0.649930	0.423199	0.022*	
C6	0.34705 (8)	0.59090 (5)	0.47087 (7)	0.01522 (15)	
H6A	0.318689	0.541383	0.450425	0.018*	
C6A	0.37463 (8)	0.60180 (5)	0.58913 (7)	0.01364 (14)	
H6AA	0.300307	0.606996	0.630059	0.016*	
C7	0.46800 (8)	0.54992 (5)	0.64181 (7)	0.01394 (14)	
H7A	0.451157	0.497142	0.631813	0.017*	
C7A	0.48436 (8)	0.57402 (5)	0.75456 (7)	0.01427 (14)	
C8	0.44763 (9)	0.54734 (5)	0.84856 (7)	0.01733 (16)	
H8A	0.402374	0.503863	0.852077	0.021*	
C9	0.47929 (9)	0.58649 (5)	0.93883 (7)	0.01878 (17)	
H9A	0.457904	0.568103	1.004751	0.023*	
C10	0.54120 (9)	0.65153 (5)	0.93385 (7)	0.01802 (16)	
H10A	0.559234	0.677754	0.995979	0.022*	
C11	0.57744 (8)	0.67895 (5)	0.83794 (7)	0.01554 (15)	
H11A	0.619080	0.723684	0.833689	0.019*	
C11A	0.55031 (8)	0.63849 (5)	0.75004 (7)	0.01308 (14)	
C11B	0.57023 (7)	0.64936 (4)	0.63521 (6)	0.01173 (13)	
C11C	0.44891 (7)	0.67267 (4)	0.58601 (6)	0.01203 (13)	
H11B	0.410997	0.713740	0.623614	0.014*	
C21	0.73244 (8)	0.63896 (5)	0.44464 (7)	0.01377 (14)	
H21A	0.796779	0.620964	0.492774	0.017*	
H21B	0.667561	0.602337	0.443280	0.017*	
C22	0.78218 (8)	0.64321 (5)	0.33735 (7)	0.01362 (14)	
C23	0.90600 (8)	0.64983 (5)	0.32688 (7)	0.01655 (15)	
H23A	0.956202	0.657652	0.386914	0.020*	
C24	0.95663 (10)	0.64511 (5)	0.22957 (8)	0.02055 (18)	
H24A	1.040992	0.649480	0.223331	0.025*	
C25	0.88364 (11)	0.63400 (5)	0.14165 (8)	0.02207 (19)	
H25A	0.917969	0.630847	0.075074	0.026*	
C26	0.76053 (11)	0.62749 (6)	0.15095 (8)	0.02329 (19)	
H26A	0.710660	0.620084	0.090611	0.028*	
C27	0.70971 (9)	0.63177 (6)	0.24837 (8)	0.01922 (17)	
H27A	0.625384	0.626885	0.254281	0.023*	
C31	0.77333 (8)	0.77043 (5)	0.47355 (7)	0.01412 (14)	
H31A	0.854007	0.752709	0.495500	0.017*	
H31B	0.774954	0.781084	0.398112	0.017*	
C32	0.74928 (8)	0.83997 (5)	0.53068 (7)	0.01367 (14)	
C33	0.80181 (9)	0.85231 (5)	0.62951 (7)	0.01728 (16)	
H33A	0.845512	0.814676	0.663914	0.021*	
C34	0.79037 (10)	0.91940 (6)	0.67762 (8)	0.02248 (19)	
H34A	0.826658	0.927443	0.744486	0.027*	
C35	0.72616 (11)	0.97452 (6)	0.62822 (9)	0.0249 (2)	
H35A	0.718829	1.020368	0.660952	0.030*	
C36	0.67257 (10)	0.96247 (5)	0.53069 (9)	0.02229 (19)	
H36A	0.627312	0.999915	0.497391	0.027*	
C37	0.68486 (9)	0.89567 (5)	0.48138 (7)	0.01687 (16)	
H37A	0.649310	0.888096	0.414119	0.020*	
N2	0.68218 (6)	0.70948 (4)	0.49050 (6)	0.01197 (12)	
O12	0.58114 (6)	0.57491 (3)	0.60106 (5)	0.01313 (11)	
O13	0.45803 (6)	0.61386 (3)	0.42482 (5)	0.01393 (11)	
C12	0.98295 (10)	0.44106 (6)	0.32533 (9)	0.0245 (2)	
O1	1.07300 (8)	0.32602 (6)	0.24533 (7)	0.0354 (2)	
O2	1.00070 (9)	0.31955 (5)	0.42121 (6)	0.03010 (18)	
O3	0.85862 (7)	0.32820 (4)	0.27351 (6)	0.02074 (14)	
F1	0.96005 (9)	0.47174 (5)	0.23305 (7)	0.0473 (2)	
F2	0.90315 (7)	0.46548 (4)	0.39205 (8)	0.0396 (2)	
F3	1.09111 (7)	0.46368 (5)	0.35900 (7)	0.03702 (18)	
S1	0.97774 (2)	0.34243 (2)	0.31472 (2)	0.01773 (5)	

### Atomic displacement parameters (Å^2^)

**Table:**

	*U*^11^	*U*^22^	*U*^33^	*U*^12^	*U*^13^	*U*^23^
C1	0.0115 (3)	0.0134 (3)	0.0132 (3)	−0.0015 (3)	−0.0003 (3)	0.0007 (3)
C3	0.0111 (3)	0.0128 (3)	0.0155 (3)	0.0004 (3)	−0.0014 (3)	0.0016 (3)
C3A	0.0103 (3)	0.0116 (3)	0.0153 (3)	0.0002 (3)	−0.0011 (3)	−0.0009 (3)
C4	0.0117 (3)	0.0177 (4)	0.0183 (4)	0.0022 (3)	−0.0029 (3)	−0.0004 (3)
C5	0.0113 (3)	0.0219 (4)	0.0207 (4)	−0.0005 (3)	−0.0027 (3)	−0.0017 (3)
C6	0.0113 (3)	0.0155 (4)	0.0188 (4)	−0.0029 (3)	0.0003 (3)	−0.0024 (3)
C6A	0.0110 (3)	0.0125 (3)	0.0175 (3)	−0.0014 (3)	0.0014 (3)	−0.0012 (3)
C7	0.0128 (3)	0.0113 (3)	0.0179 (4)	−0.0013 (3)	0.0031 (3)	−0.0003 (3)
C7A	0.0130 (3)	0.0129 (3)	0.0170 (3)	0.0009 (3)	0.0019 (3)	0.0011 (3)
C8	0.0176 (4)	0.0152 (4)	0.0194 (4)	0.0019 (3)	0.0045 (3)	0.0039 (3)
C9	0.0194 (4)	0.0204 (4)	0.0168 (4)	0.0063 (3)	0.0041 (3)	0.0038 (3)
C10	0.0183 (4)	0.0201 (4)	0.0156 (4)	0.0057 (3)	−0.0005 (3)	0.0002 (3)
C11	0.0143 (4)	0.0157 (4)	0.0164 (3)	0.0014 (3)	−0.0020 (3)	0.0000 (3)
C11A	0.0119 (3)	0.0128 (3)	0.0145 (3)	0.0009 (3)	0.0004 (3)	0.0010 (3)
C11B	0.0106 (3)	0.0100 (3)	0.0146 (3)	0.0001 (2)	0.0004 (3)	−0.0001 (2)
C11C	0.0101 (3)	0.0115 (3)	0.0145 (3)	0.0005 (2)	0.0001 (3)	−0.0010 (2)
C21	0.0133 (3)	0.0116 (3)	0.0164 (3)	0.0013 (3)	0.0016 (3)	−0.0003 (3)
C22	0.0131 (3)	0.0122 (3)	0.0156 (3)	0.0001 (3)	0.0006 (3)	−0.0008 (3)
C23	0.0140 (4)	0.0171 (4)	0.0187 (4)	−0.0012 (3)	0.0024 (3)	−0.0025 (3)
C24	0.0208 (4)	0.0178 (4)	0.0236 (4)	−0.0025 (3)	0.0082 (3)	−0.0025 (3)
C25	0.0333 (5)	0.0157 (4)	0.0177 (4)	−0.0007 (4)	0.0074 (4)	−0.0001 (3)
C26	0.0309 (5)	0.0227 (4)	0.0160 (4)	0.0038 (4)	−0.0032 (4)	−0.0018 (3)
C27	0.0177 (4)	0.0209 (4)	0.0188 (4)	0.0013 (3)	−0.0029 (3)	−0.0030 (3)
C31	0.0127 (3)	0.0125 (3)	0.0172 (3)	−0.0023 (3)	0.0015 (3)	0.0003 (3)
C32	0.0138 (3)	0.0122 (3)	0.0151 (3)	−0.0025 (3)	0.0012 (3)	0.0001 (3)
C33	0.0188 (4)	0.0173 (4)	0.0157 (3)	−0.0055 (3)	−0.0004 (3)	0.0015 (3)
C34	0.0297 (5)	0.0222 (4)	0.0158 (4)	−0.0099 (4)	0.0042 (3)	−0.0037 (3)
C35	0.0310 (5)	0.0168 (4)	0.0273 (5)	−0.0052 (4)	0.0096 (4)	−0.0062 (3)
C36	0.0229 (5)	0.0139 (4)	0.0303 (5)	0.0011 (3)	0.0043 (4)	0.0003 (3)
C37	0.0173 (4)	0.0136 (4)	0.0196 (4)	−0.0010 (3)	−0.0005 (3)	0.0015 (3)
N2	0.0105 (3)	0.0110 (3)	0.0144 (3)	−0.0006 (2)	−0.0001 (2)	0.0003 (2)
O12	0.0115 (3)	0.0100 (2)	0.0181 (3)	0.0002 (2)	0.0031 (2)	−0.0004 (2)
O13	0.0125 (3)	0.0128 (3)	0.0165 (3)	−0.0017 (2)	0.0011 (2)	−0.0028 (2)
C12	0.0229 (5)	0.0225 (5)	0.0279 (5)	−0.0046 (4)	−0.0027 (4)	0.0011 (4)
O1	0.0193 (4)	0.0552 (6)	0.0318 (4)	0.0077 (4)	0.0022 (3)	−0.0185 (4)
O2	0.0398 (5)	0.0281 (4)	0.0217 (3)	0.0043 (3)	−0.0110 (3)	0.0035 (3)
O3	0.0173 (3)	0.0217 (3)	0.0230 (3)	0.0002 (3)	−0.0032 (3)	−0.0028 (3)
F1	0.0658 (6)	0.0315 (4)	0.0432 (5)	−0.0135 (4)	−0.0198 (4)	0.0176 (3)
F2	0.0332 (4)	0.0242 (4)	0.0618 (5)	0.0008 (3)	0.0086 (4)	−0.0171 (3)
F3	0.0293 (4)	0.0414 (4)	0.0401 (4)	−0.0172 (3)	−0.0037 (3)	−0.0041 (3)
S1	0.01597 (10)	0.02037 (11)	0.01662 (10)	0.00469 (8)	−0.00298 (7)	−0.00322 (7)

### Geometric parameters (Å, º)

**Table:**

C1—C11B	1.5014 (12)	C21—C22	1.5043 (12)
C1—N2	1.5132 (11)	C21—N2	1.5421 (11)
C1—H1A	0.9900	C21—H21A	0.9900
C1—H1B	0.9900	C21—H21B	0.9900
C3—C3A	1.5077 (12)	C22—C27	1.3956 (13)
C3—N2	1.5198 (11)	C22—C23	1.3967 (13)
C3—H3A	0.9900	C23—C24	1.3909 (13)
C3—H3B	0.9900	C23—H23A	0.9500
C3A—O13	1.4427 (10)	C24—C25	1.3870 (15)
C3A—C4	1.5231 (12)	C24—H24A	0.9500
C3A—C11C	1.5514 (12)	C25—C26	1.3869 (17)
C4—C5	1.3339 (13)	C25—H25A	0.9500
C4—H4A	0.9500	C26—C27	1.3925 (15)
C5—C6	1.5253 (14)	C26—H26A	0.9500
C5—H5A	0.9500	C27—H27A	0.9500
C6—O13	1.4515 (11)	C31—C32	1.5074 (12)
C6—C6A	1.5543 (13)	C31—N2	1.5365 (11)
C6—H6A	1.0000	C31—H31A	0.9900
C6A—C11C	1.5500 (12)	C31—H31B	0.9900
C6A—C7	1.5544 (13)	C32—C37	1.3952 (13)
C6A—H6AA	1.0000	C32—C33	1.4007 (13)
C7—O12	1.4560 (11)	C33—C34	1.3921 (14)
C7—C7A	1.5214 (13)	C33—H33A	0.9500
C7—H7A	1.0000	C34—C35	1.3869 (17)
C7A—C8	1.3787 (13)	C34—H34A	0.9500
C7A—C11A	1.4014 (12)	C35—C36	1.3897 (16)
C8—C9	1.4029 (14)	C35—H35A	0.9500
C8—H8A	0.9500	C36—C37	1.3955 (14)
C9—C10	1.3876 (15)	C36—H36A	0.9500
C9—H9A	0.9500	C37—H37A	0.9500
C10—C11	1.4037 (13)	C12—F1	1.3318 (14)
C10—H10A	0.9500	C12—F2	1.3333 (14)
C11—C11A	1.3800 (12)	C12—F3	1.3357 (13)
C11—H11A	0.9500	C12—S1	1.8271 (12)
C11A—C11B	1.5125 (12)	O1—S1	1.4404 (9)
C11B—O12	1.4494 (10)	O2—S1	1.4476 (8)
C11B—C11C	1.5369 (12)	O3—S1	1.4387 (8)
C11C—H11B	1.0000		
			
C11B—C1—N2	114.03 (7)	C6A—C11C—H11B	113.2
C11B—C1—H1A	108.7	C3A—C11C—H11B	113.2
N2—C1—H1A	108.7	C22—C21—N2	117.07 (7)
C11B—C1—H1B	108.7	C22—C21—H21A	108.0
N2—C1—H1B	108.7	N2—C21—H21A	108.0
H1A—C1—H1B	107.6	C22—C21—H21B	108.0
C3A—C3—N2	114.76 (7)	N2—C21—H21B	108.0
C3A—C3—H3A	108.6	H21A—C21—H21B	107.3
N2—C3—H3A	108.6	C27—C22—C23	118.90 (9)
C3A—C3—H3B	108.6	C27—C22—C21	121.51 (8)
N2—C3—H3B	108.6	C23—C22—C21	119.12 (8)
H3A—C3—H3B	107.6	C24—C23—C22	120.75 (9)
O13—C3A—C3	113.64 (7)	C24—C23—H23A	119.6
O13—C3A—C4	101.07 (7)	C22—C23—H23A	119.6
C3—C3A—C4	118.43 (7)	C25—C24—C23	119.83 (10)
O13—C3A—C11C	102.99 (6)	C25—C24—H24A	120.1
C3—C3A—C11C	114.39 (7)	C23—C24—H24A	120.1
C4—C3A—C11C	104.37 (7)	C26—C25—C24	119.99 (9)
C5—C4—C3A	104.92 (8)	C26—C25—H25A	120.0
C5—C4—H4A	127.5	C24—C25—H25A	120.0
C3A—C4—H4A	127.5	C25—C26—C27	120.28 (9)
C4—C5—C6	106.07 (8)	C25—C26—H26A	119.9
C4—C5—H5A	127.0	C27—C26—H26A	119.9
C6—C5—H5A	127.0	C26—C27—C22	120.25 (9)
O13—C6—C5	100.79 (7)	C26—C27—H27A	119.9
O13—C6—C6A	102.46 (7)	C22—C27—H27A	119.9
C5—C6—C6A	106.05 (7)	C32—C31—N2	115.24 (7)
O13—C6—H6A	115.3	C32—C31—H31A	108.5
C5—C6—H6A	115.3	N2—C31—H31A	108.5
C6A—C6—H6A	115.3	C32—C31—H31B	108.5
C11C—C6A—C6	100.00 (7)	N2—C31—H31B	108.5
C11C—C6A—C7	100.40 (7)	H31A—C31—H31B	107.5
C6—C6A—C7	117.10 (7)	C37—C32—C33	119.13 (8)
C11C—C6A—H6AA	112.6	C37—C32—C31	120.24 (8)
C6—C6A—H6AA	112.6	C33—C32—C31	120.30 (8)
C7—C6A—H6AA	112.6	C34—C33—C32	120.40 (9)
O12—C7—C7A	99.81 (7)	C34—C33—H33A	119.8
O12—C7—C6A	102.95 (7)	C32—C33—H33A	119.8
C7A—C7—C6A	107.06 (7)	C35—C34—C33	120.19 (9)
O12—C7—H7A	115.1	C35—C34—H34A	119.9
C7A—C7—H7A	115.1	C33—C34—H34A	119.9
C6A—C7—H7A	115.1	C34—C35—C36	119.79 (9)
C8—C7A—C11A	120.71 (8)	C34—C35—H35A	120.1
C8—C7A—C7	134.42 (8)	C36—C35—H35A	120.1
C11A—C7A—C7	104.86 (7)	C35—C36—C37	120.37 (10)
C7A—C8—C9	117.80 (9)	C35—C36—H36A	119.8
C7A—C8—H8A	121.1	C37—C36—H36A	119.8
C9—C8—H8A	121.1	C32—C37—C36	120.11 (9)
C10—C9—C8	121.38 (9)	C32—C37—H37A	119.9
C10—C9—H9A	119.3	C36—C37—H37A	119.9
C8—C9—H9A	119.3	C1—N2—C3	112.75 (7)
C9—C10—C11	120.66 (9)	C1—N2—C31	108.23 (6)
C9—C10—H10A	119.7	C3—N2—C31	108.19 (6)
C11—C10—H10A	119.7	C1—N2—C21	107.41 (6)
C11A—C11—C10	117.51 (9)	C3—N2—C21	111.80 (6)
C11A—C11—H11A	121.2	C31—N2—C21	108.33 (7)
C10—C11—H11A	121.2	C11B—O12—C7	96.31 (6)
C11—C11A—C7A	121.86 (8)	C3A—O13—C6	95.89 (6)
C11—C11A—C11B	133.76 (8)	F1—C12—F2	108.40 (11)
C7A—C11A—C11B	104.27 (7)	F1—C12—F3	107.55 (10)
O12—C11B—C1	115.03 (7)	F2—C12—F3	107.45 (9)
O12—C11B—C11A	100.71 (6)	F1—C12—S1	110.65 (8)
C1—C11B—C11A	117.03 (7)	F2—C12—S1	111.39 (8)
O12—C11B—C11C	102.84 (6)	F3—C12—S1	111.23 (8)
C1—C11B—C11C	113.06 (7)	O3—S1—O1	115.08 (5)
C11A—C11B—C11C	106.53 (7)	O3—S1—O2	115.39 (5)
C11B—C11C—C6A	102.54 (7)	O1—S1—O2	114.31 (6)
C11B—C11C—C3A	111.64 (7)	O3—S1—C12	103.67 (5)
C6A—C11C—C3A	102.16 (6)	O1—S1—C12	103.51 (6)
C11B—C11C—H11B	113.2	O2—S1—C12	102.48 (5)
			
N2—C3—C3A—O13	−72.16 (9)	O13—C3A—C11C—C6A	−31.68 (8)
N2—C3—C3A—C4	169.47 (7)	C3—C3A—C11C—C6A	−155.48 (7)
N2—C3—C3A—C11C	45.73 (10)	C4—C3A—C11C—C6A	73.54 (8)
O13—C3A—C4—C5	34.74 (9)	N2—C21—C22—C27	−89.63 (10)
C3—C3A—C4—C5	159.53 (8)	N2—C21—C22—C23	98.39 (10)
C11C—C3A—C4—C5	−71.91 (9)	C27—C22—C23—C24	−0.03 (14)
C3A—C4—C5—C6	−1.85 (10)	C21—C22—C23—C24	172.16 (9)
C4—C5—C6—O13	−31.30 (9)	C22—C23—C24—C25	0.27 (15)
C4—C5—C6—C6A	75.15 (9)	C23—C24—C25—C26	−0.12 (15)
O13—C6—C6A—C11C	38.02 (8)	C24—C25—C26—C27	−0.26 (16)
C5—C6—C6A—C11C	−67.23 (8)	C25—C26—C27—C22	0.50 (16)
O13—C6—C6A—C7	−69.21 (9)	C23—C22—C27—C26	−0.35 (14)
C5—C6—C6A—C7	−174.45 (7)	C21—C22—C27—C26	−172.35 (9)
C11C—C6A—C7—O12	−34.81 (8)	N2—C31—C32—C37	−94.11 (10)
C6—C6A—C7—O12	72.18 (9)	N2—C31—C32—C33	92.59 (10)
C11C—C6A—C7—C7A	69.87 (8)	C37—C32—C33—C34	−0.33 (14)
C6—C6A—C7—C7A	176.86 (7)	C31—C32—C33—C34	173.05 (9)
O12—C7—C7A—C8	−147.50 (10)	C32—C33—C34—C35	0.37 (15)
C6A—C7—C7A—C8	105.58 (11)	C33—C34—C35—C36	0.38 (16)
O12—C7—C7A—C11A	34.01 (8)	C34—C35—C36—C37	−1.18 (16)
C6A—C7—C7A—C11A	−72.91 (8)	C33—C32—C37—C36	−0.47 (14)
C11A—C7A—C8—C9	−0.49 (13)	C31—C32—C37—C36	−173.84 (9)
C7—C7A—C8—C9	−178.79 (9)	C35—C36—C37—C32	1.22 (15)
C7A—C8—C9—C10	2.63 (14)	C11B—C1—N2—C3	50.16 (9)
C8—C9—C10—C11	−2.06 (14)	C11B—C1—N2—C31	169.79 (7)
C9—C10—C11—C11A	−0.70 (13)	C11B—C1—N2—C21	−73.44 (8)
C10—C11—C11A—C7A	2.86 (13)	C3A—C3—N2—C1	−46.62 (9)
C10—C11—C11A—C11B	178.34 (9)	C3A—C3—N2—C31	−166.28 (7)
C8—C7A—C11A—C11	−2.30 (14)	C3A—C3—N2—C21	74.51 (9)
C7—C7A—C11A—C11	176.44 (8)	C32—C31—N2—C1	−53.16 (9)
C8—C7A—C11A—C11B	−178.94 (8)	C32—C31—N2—C3	69.30 (9)
C7—C7A—C11A—C11B	−0.19 (9)	C32—C31—N2—C21	−169.32 (7)
N2—C1—C11B—O12	65.09 (9)	C22—C21—N2—C1	−163.77 (7)
N2—C1—C11B—C11A	−176.98 (7)	C22—C21—N2—C3	72.05 (9)
N2—C1—C11B—C11C	−52.64 (9)	C22—C21—N2—C31	−47.07 (9)
C11—C11A—C11B—O12	149.99 (10)	C1—C11B—O12—C7	−178.83 (7)
C7A—C11A—C11B—O12	−33.96 (8)	C11A—C11B—O12—C7	54.39 (7)
C11—C11A—C11B—C1	24.55 (14)	C11C—C11B—O12—C7	−55.49 (7)
C7A—C11A—C11B—C1	−159.40 (7)	C7A—C7—O12—C11B	−53.98 (7)
C11—C11A—C11B—C11C	−103.03 (11)	C6A—C7—O12—C11B	56.21 (7)
C7A—C11A—C11B—C11C	73.01 (8)	C3—C3A—O13—C6	179.68 (7)
O12—C11B—C11C—C6A	33.75 (8)	C4—C3A—O13—C6	−52.35 (7)
C1—C11B—C11C—C6A	158.40 (7)	C11C—C3A—O13—C6	55.39 (7)
C11A—C11B—C11C—C6A	−71.70 (8)	C5—C6—O13—C3A	50.91 (7)
O12—C11B—C11C—C3A	−74.93 (8)	C6A—C6—O13—C3A	−58.38 (7)
C1—C11B—C11C—C3A	49.72 (9)	F1—C12—S1—O3	−57.94 (9)
C11A—C11B—C11C—C3A	179.62 (7)	F2—C12—S1—O3	62.72 (9)
C6—C6A—C11C—C11B	−119.49 (7)	F3—C12—S1—O3	−177.43 (8)
C7—C6A—C11C—C11B	0.69 (8)	F1—C12—S1—O1	62.53 (10)
C6—C6A—C11C—C3A	−3.75 (8)	F2—C12—S1—O1	−176.81 (8)
C7—C6A—C11C—C3A	116.43 (7)	F3—C12—S1—O1	−56.96 (9)
O13—C3A—C11C—C11B	77.25 (8)	F1—C12—S1—O2	−178.33 (9)
C3—C3A—C11C—C11B	−46.55 (9)	F2—C12—S1—O2	−57.67 (9)
C4—C3A—C11C—C11B	−177.53 (7)	F3—C12—S1—O2	62.18 (9)

### Hydrogen-bond geometry (Å, º)

*Cg*10 is the centroid of the C32–C37 ring.

**Table:**

*D*—H···*A*	*D*—H	H···*A*	*D*···*A*	*D*—H···*A*
C1—H1*A*···O1^i^	0.99	2.49	3.4043 (13)	154
C6—H6*A*···O12^ii^	1.00	2.52	3.3040 (11)	135
C6*A*—H6*AA*···O3^ii^	1.00	2.50	3.4407 (12)	157
C7—H7*A*···O13^ii^	1.00	2.41	3.2563 (11)	142
C21—H21*B*···O12	0.99	2.33	2.9151 (11)	117
C21—H21*B*···O13	0.99	2.35	3.0974 (11)	132
C31—H31*A*···O2^i^	0.99	2.33	3.2751 (13)	159
C9—H9*A*···*Cg*10^iii^	0.95	2.71	3.2958 (11)	121

Symmetry codes: (i) −*x*+2, −*y*+1, −*z*+1; (ii) −*x*+1, −*y*+1, −*z*+1; (iii) *x*−1/2, −*y*+3/2, *z*+1/2.
